# Beyond the Implant: Towards a Predictive, Preventive, Personalized and Participatory Approach to Patient-Centered Implant Dentistry—A Narrative Review

**DOI:** 10.3390/jcm15207795

**Published:** 2026-10-09

**Authors:** Pascal Valentini, Raphaëlle Contencin, Giancarlo Bianca, Laura Constantini

**Affiliations:** Department of Oral Implantology, University of Corsica Pascal Paoli, 20250 Corte, France; dr.contencin@outlook.fr (R.C.); bianca.cipe@orange.fr (G.B.); docteurlauraconstantini@gmail.com (L.C.)

**Keywords:** dental implants, 4P medicine, predictive dentistry, peri-implantitis prevention, shared decision-making, patient-reported outcomes, artificial intelligence, osseointegration

## Abstract

Contemporary implant dentistry achieves remarkable survival rates, yet the rising prevalence of peri-implantitis and increasing patient complexity reveal the limits of a technically centered model. This narrative review synthesizes current evidence supporting the four pillars of the 4P framework—predictive, preventive, personalized, and participatory—and proposes an integrated patient-centered clinical algorithm for implant dentistry. Throughout this review, “4P” is used as the preferred designation; “P4 medicine” appears only in direct citation of original systems-medicine sources. A narrative literature search was conducted in PubMed, Scopus, and the Cochrane Library (January 2000 to June 2026). Reporting followed Scale for the Assessment of Narrative Review Articles (SANRA) Predictive tools—resonance frequency analysis, genomic biomarkers [investigational], artificial intelligence algorithms, and radiomic analysis—contribute to individual risk stratification, with variable levels of clinical validation. Preventive strategies anchored in a four-level continuum are associated with a peri-implantitis frequency of 18% in maintenance-compliant patients versus 43.9% in non-compliant patients, illustrating the measurable clinical impact of the preventive pillar. Personalized approaches optimize osseointegration according to each patient’s biological profile. Participatory implantology, through shared decision-making and patient-reported outcome measures, improves the therapeutic alliance and long-term adherence. The 4P model constitutes a coherent and clinically applicable conceptual framework. Its novel contribution is a sequential decision-making logic distinguishing pre-placement gate-keeping from post-placement dynamic pillar reorientation, providing clinicians with a structured conceptual framework applicable at each phase of implant treatment.

## 1. Introduction

Since Per-Ingvar Brånemark first described osseointegration in the 1960s, implant dentistry has established itself as one of the most evidence-based disciplines in modern oral medicine [1,2]. Ten-year implant survival rates consistently exceed 95% under controlled conditions [3], and implant-supported restorations have become the reference standard for replacing missing teeth across a wide range of clinical situations.

Yet this technical success is increasingly overshadowed by a growing biological challenge. Peri-implant mucositis affects approximately 43% of implant-bearing patients, and peri-implantitis—characterized by progressive bone loss—affects between 20% and 56% of patients depending on diagnostic criteria and follow-up duration [4,5]. These figures reveal the limitations of a model that has historically privileged surgical precision over individual biological risk management, long-term maintenance, and patient engagement.

**Figure 1:**
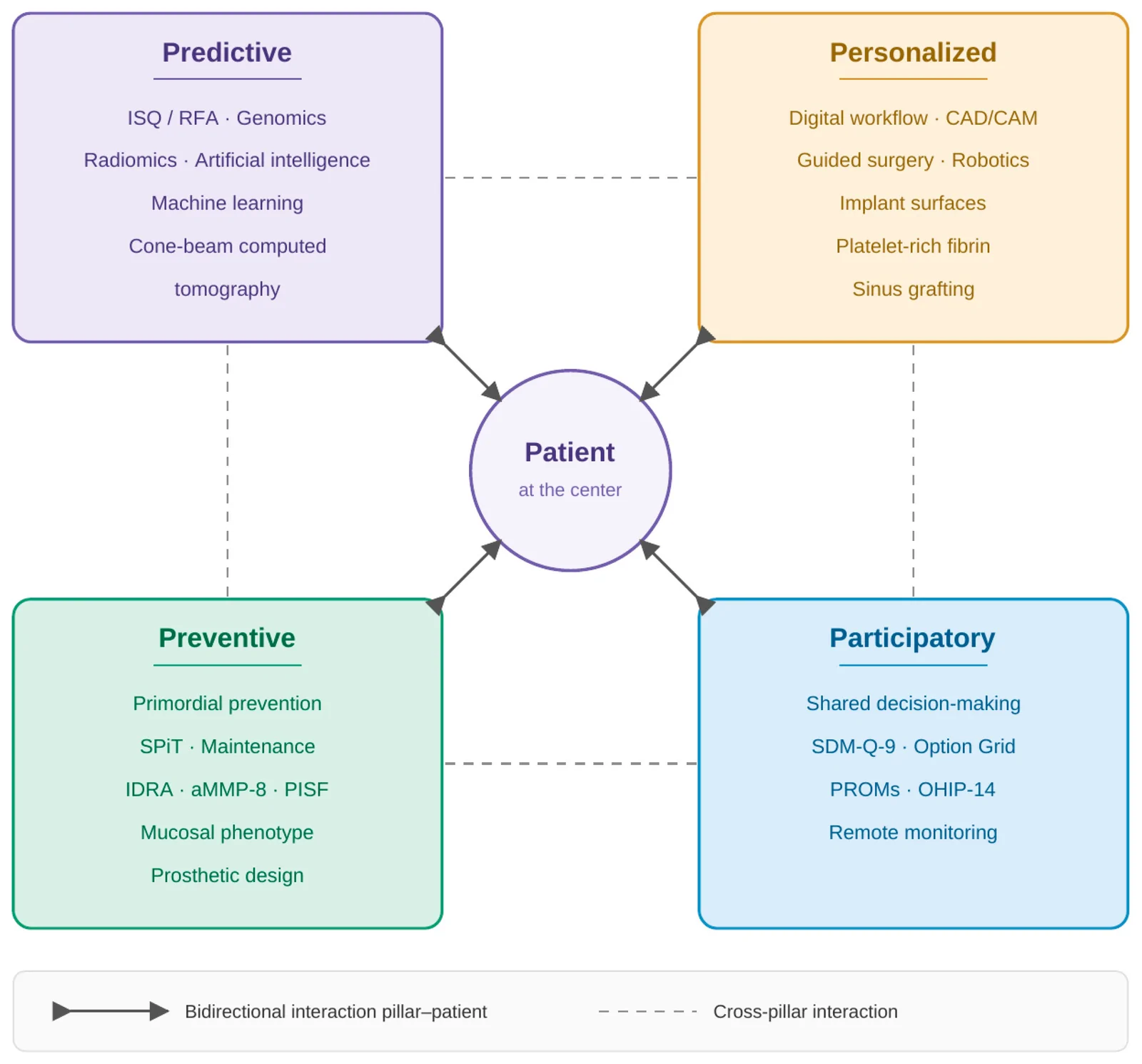
Interrelations between the four pillars of the 4P model in implant dentistry. Solid bidirectional arrows represent direct interactions between each pillar and the patient. Dashed lines indicate cross-pillar interactions. Abbreviations: aMMP-8, active matrix metalloproteinase-8; CAD/CAM, computer-aided design/computer-aided manufacturing; IDRA, Implant Disease Risk Assessment; ISQ, implant stability quotient; OHIP-14, Oral Health Impact Profile-14; PISF, peri-implant sulcular fluid; PROMs, patient-reported outcome measures; RFA, resonance frequency analysis; SDM-Q-9, 9-item Shared Decision Making Questionnaire; SPiT, supportive peri-implant therapy.

The true success of a dental implant is not measured solely by initial osseointegration, but at 10, 15, or 20 years, when the implant remains in place, functional, and surrounded by healthy tissues. It is in this long-term perspective that the 4P framework—predictive, preventive, personalized, and participatory—emerges as a structuring model for contemporary implant dentistry (Figure 1).

Originally coined by Hood and Flores in systems medicine [6], applied to surgery by Slim et al. [7], and subsequently transposed to periodontology by Bartold and Ivanovski [8], the 4P concept (designated throughout this review as “4P” rather than “P4”) has demonstrated its potential to transform health care by quantifying individual well-being and personalizing management. In dentistry, Beck et al. described the shift toward precision dentistry integrating individual genetic, environmental, and clinical variability [9], while Fuglsig et al. noted that advanced artificial intelligence technologies will enable personalized, predictive, preventive, and participatory dentistry [10].

The present narrative review synthesizes the current evidence supporting each pillar of the 4P model as applied to implant dentistry and proposes an integrated patient-centered conceptual framework. A novel contribution is a sequential decision-making logic distinguishing the pre-placement phase (go/no-go decision) from the post-placement phase (dynamic pillar reorientation). A further original contribution is the explicit distinction between early implant loss (EIL) and peri-implantitis as two biologically distinct entities requiring different predictive tools, different prevention strategies, and different clinical responses.

## 2. Materials and Methods

A narrative literature search was conducted in PubMed, Scopus, and the Cochrane Library, covering publications from January 2000 to June 2026. Representative search strings included: (“dental implants” OR “osseointegration”) AND (“predictive medicine” OR “risk stratification”); (“peri-implantitis”) AND (“prevention” OR “maintenance” OR “biomarkers”); (“dental implants”) AND (“shared decision making” OR “patient-reported outcomes”). Evidence was mapped to the four pillars: risk prediction and biomarkers → Predictive; prevention guidelines and maintenance protocols → Preventive; technology and materials → Personalized; SDM and PROMs → Participatory. Numerical thresholds were included in the algorithm only when derived from guideline-endorsed consensus or validated in ≥2 independent cohorts. Articles in English and French were included. Given the narrative design, no formal inclusion or exclusion criteria were applied. This review was conducted in accordance with SANRA. The clinical algorithm presented in Tables 2 and 3 should be interpreted as a conceptual framework integrating current evidence with expert synthesis, not as a validated evidence-based protocol. A three-tier evidence classification is applied throughout Tables 2 and 3: ✓ Established; ◑ Promising; ○ Investigational.

## 3. Results

### 3.1. Predictive Implantology

#### 3.1.1. Clinical Risk Factors and Systemic Conditions

The epidemiologic determinants of implant failure have been extensively studied. A history of treated periodontitis remains the most consistent risk indicator [11,12]. Smoking increases implant failure risk with an odds ratio exceeding 12 in some cohorts [13]. Uncontrolled type 2 diabetes (HbA1c > 8%) and obesity have been formally recognized as systemic risk factors in the most recent AO/AAP consensus [12]. The HbA1c thresholds in Table 1 (<7% desirable; >8% systemic risk factor) are derived from the 2025 AO/AAP consensus [12], not from the authors’ independent recommendations. Table 1 summarizes the principal pre-implant risk factors and the management strategy associated with each.

#### 3.1.2. Three-Dimensional Imaging, Digital Planning, and Radiomic Biomarkers

CBCT and digital planning software have fundamentally transformed implant outcome prediction [14]. CBCT attenuation values are not true Hounsfield Units: they are apparatus-specific, affected by beam-hardening artifacts, and not transferable between devices. The correlation between CBCT-derived attenuation values and primary ISQ (r = 0.719) should therefore be interpreted as a relative, device-specific association rather than an absolute predictive metric. Resonance frequency analysis and ISQ provide noninvasive monitoring of osseointegration in real time [15]. AI-based automatic segmentation of the maxillary sinus enables volumetric analysis in under 1 min [16]. AI-based peri-implant bone mineral density mapping shows strong correlation with primary stability [17]. CBCT-based homogenized finite element analysis enables millimetric prediction of primary stability [18,19]. Radiomic biomarkers extracted from intraoral radiographs can predict physiologic bone remodeling [20].

#### 3.1.3. Primary Stability and Osseointegration: Predictive Determinants

Early implant loss (EIL) and peri-implantitis are two biologically distinct entities with different determinants, different time windows, and different clinical expressions. EIL—defined as absence of rigid fixation before prosthetic loading—typically occurs during the first weeks after placement, before bacterial biofilm colonization reaches the threshold for peri-implantitis [15]. Conflating these two entities weakens both the diagnostic approach and the preventive strategy.

Insertion torque and ISQ at placement constitute complementary objective measures: an ISQ of 65 or above is generally associated with favorable osseointegration conditions, whereas below 60 the risk of secondary instability is significantly increased and immediate loading is contraindicated [15,21]. ISQ values must be interpreted in the context of implant macrogeometry: highly tapered designs with aggressive thread profiles may generate elevated ISQ through excessive cortical compression rather than biological bonding, carrying risk of ischemia and compaction necrosis. Pre-placement predictive assessment relies on imaging-derived bone density and clinical risk factors, not on ISQ, which begins at implant placement. Secondary stability develops progressively over 4 to 12 weeks [22]. Serial ISQ monitoring at weeks 2, 4, and 8 enables detection of anomalous patterns; a persistently declining ISQ beyond week 4 is an early warning signal of EIL, preceding any radiographic evidence [15]. ISQ monitoring during W2–W4 must be conducted without abutment removal to avoid disruption of the developing peri-implant mucosal seal; repeated SmartPeg attachment during the critical healing period disrupts the fragile mucosal seal and induces marginal crestal bone resorption. ISQ re-measurement is reserved for the week-8 timepoint when the mucosal seal is established.

#### 3.1.4. Genomic Risk Profiling

The concept of implantogenomics, introduced by Refai and Cochran [23], applies omics sciences to the prediction of osseointegration outcomes. Germline genetic polymorphisms representing stable preoperative risk factors include IL1B +3954 C/T, TNF -308G/A, CD14 rs2569190, and MMP-8 -799C/T [24]. Commercial genetic tests now enable chairside risk profiling before implant placement, although their translation from population-level associations to individual predictive value requires further prospective validation before routine recommendation. For this reason, genomic profiling is classified ○ (Investigational) in Table 2 and Table 3 and has been removed from the Table 3 decision algorithm as a criterion for modifying the surgical protocol.

#### 3.1.5. Artificial Intelligence and Machine Learning

Important methodological caveat: virtually all published AI models for implant outcome prediction are based on single-center retrospective datasets, carry a high overfitting risk, and have not undergone prospective multicenter external validation. The AUC values reported below therefore reflect in-sample or limited-validation performance, not established clinical predictive validity. Machine learning algorithms integrate multiple data streams to improve prognostic assessment. Recent umbrella reviews demonstrate that convolutional neural networks achieve accuracy rates exceeding 90% for implant type recognition and moderate accuracy (62–81%) for implant success prediction [25,26,27]. A random forest model reached an AUC of 0.71 for peri-implantitis prediction [28]; a web-based model achieved an AUC of 0.872 for implant failure prediction [29]; and a multimodal deep learning model reached an AUC exceeding 0.80 [30]. Most models remain monocentric and require prospective multicenter external validation [25,26]. Explainable AI approaches represent a priority research direction.

### 3.2. Preventive Implantology

#### 3.2.1. Soft Tissue Phenotype and Primary Prevention

Beyond keratinized mucosal width, supracrestal tissue height (STH)—the vertical distance from crestal bone to the mucosal margin—has emerged as an independent preventive parameter. STH below 3 mm is independently associated with greater marginal bone loss in the first year post-loading. STH measurement at implant placement is recommended in thin mucosal phenotypes; if STH is below 3 mm, connective tissue augmentation or deeper subcrestal positioning should be considered. A keratinized mucosal width below 2 mm is associated with significantly higher risk of peri-implant dehiscence and peri-implantitis [31]. The EFP S3-level guideline recommends keratinized mucosa augmentation when width is insufficient [32,33]. [Note: Table 1 uses “insufficient keratinized mucosal width (<2 mm)” rather than “unfavorable mucosal phenotype”—these concepts are not interchangeable; “mucosal phenotype” encompasses width, thickness, and biotype.]

#### 3.2.2. Prehabilitation and Four-Level Prevention Continuum

The concept of prehabilitation in implant dentistry, formalized by Carra and Bouchard [34], operationalizes primordial and primary prevention as a structured pre-implant optimization program. It should be emphasized that prehabilitation does not introduce new therapeutic actions: treatment of active periodontitis, glycemic control, and smoking cessation are fundamental Phase I periodontal obligations. The conceptual contribution is the formalization of a structured multidomain timeline with specific measurable targets and reassessment milestones for each risk domain—analogous to surgical prehabilitation in oncology. Building on the EFP S3-level guideline [32] and Carra et al. [34], preventive implantology is organized into four successive levels: primordial prevention; primary prevention; secondary prevention; and tertiary prevention.

#### 3.2.3. Chairside Biomarker Monitoring

RUNX2—the master transcription factor for osteogenesis—shows significantly higher expression at healthy osseointegrated implant sites than at peri-implantitis sites (SMD 2.58; 95% CI 1.21–3.95; *p* < 0.001), making its PISF measurement a potential marker of ongoing osseointegration quality [35]. Oral fluid biomarkers for peri-implantitis, including PISF cytokines and salivary analytes, represent non-invasive monitoring tools with growing diagnostic evidence [36]. Active matrix metalloproteinase-8 (aMMP-8) chairside point-of-care testing at 20 ng/mL has been proposed as an early warning signal [37]. An important confound: active or untreated periodontitis in the remaining dentition elevates sulcular aMMP-8 regardless of peri-implant status, potentially generating false-positive signals. Chairside biomarker monitoring of peri-implant sites is therefore only interpretable in patients with full-mouth periodontal stability. Salivary biosensors for real-time biomarker monitoring are under development [38]. The clinical translation of these biomarkers remains constrained by the absence of universally validated thresholds.

#### 3.2.4. Prosthetic Design, Supportive Therapy, and Compliance

Prosthetic design constitutes a primary preventive factor defined at the time of restoration. Evidence-based preventive prosthetic parameters include: (1) emergence profile angle below 30°; (2) platform switching with internal conical connections; (3) screw-retained preference over cement-retained restorations; and (4) embrasure design accommodating interdental brush passage. These parameters should be planned at the pre-surgical digital workflow stage and communicated to the patient during therapeutic education. Compliance with regular SPiT is associated with a peri-implantitis incidence of 18% in compliant versus 43.9% in non-compliant patients [39]. This associative finding derives from a cohort analysis and should not be generalized as the universal outcome of any prevention model, given the extreme heterogeneity in diagnostic criteria across studies. Systematic review and meta-analysis evidence demonstrates that SPiT reduces peri-implant disease risk by a factor exceeding 5 (OR 5.26; 95% CI: 2.62–10.55) [40], a finding confirmed across diverse clinical protocols [41]. Risk-stratified maintenance, operationalized through the Implant Disease Risk Assessment (IDRA) tool [42], enables individualization of recall intervals: every 3 to 4 months for high-risk patients, every 6 months for moderate-risk patients, and annually for low-risk patients [32]. Each SPiT session encompasses both supramucosal and submucosal biofilm disruption. For patients at moderate-to-high IDRA risk (probing depth ≥ 4 mm), submucosal debridement using implant-compatible instruments is included at each recall visit.

### 3.3. Personalized Implantology

Within the 4P framework, an intervention qualifies as “personalized” when the selection between available options is conditioned by a patient-specific biological, clinical, anatomical, or risk-profile characteristic, rather than applied uniformly.

#### 3.3.1. Surgical Guidance

Static computer-assisted implant surgery (sCAIS), dynamic navigation (dCAIS), and robotic surgery constitute a precision gradient quantified in a network meta-analysis [43]. Robotic surgery and dynamic navigation achieve significantly lower apical deviation than freehand technique [43,44]. The precision advantage is operator-dependent: learning curve effects, fiducial marker instability, and registration error introduce variability not captured by mean deviation statistics. The performance metrics reported reflect experienced operators under controlled conditions.

#### 3.3.2. Personalization of the Therapeutic Timeline and Loading Protocol

Immediate loading is possible when ISQ ≥ 65 and insertion torque ≥ 35 Ncm AND: (1) antagonist environment is favorable (natural dentition with normal occlusal scheme, or complete denture); (2) parafunctional habits are absent or controlled; (3) the provisional restoration is in slight infraocclusion with minimal lateral guidance contacts. Implant-supported antagonist restorations or natural dentition with bruxism/clenching represent relative contraindications to immediate loading [45].

#### 3.3.3. Implant Surfaces and Biological Personalization

Hydrophilic modified SLA surfaces (mSLA) achieve bone-to-implant contact of approximately 50% in animal models, compared with 40–42% for conventional SLA [46]. Nanostructured surfaces on TiZr alloys demonstrate greater osseointegration potential than conventional SLA in osteoporotic bone models [47], providing an additional personalization option in patients with compromised bone quality. Animal-model data require qualification: performance may not translate directly to clinical outcomes in human patients with compromised bone quality. Furthermore, rough hydrophilic surfaces are more susceptible to pathogenic biofilm colonization and present specific decontamination challenges in established peri-implantitis. The optimal roughness range (Sa 1.0–2.0 µm) maximizes osseointegration while limiting biofilm accumulation [48,49] in patients with compromised bone quality. In compromised osseointegration animal models, modified surfaces demonstrate differential performance [50].

#### 3.3.4. Maxillary Sinus Floor Augmentation

Maxillary sinus augmentation by the lateral window approach remains the reference standard for the atrophic posterior maxilla [51,52,53,54,55,56,57]. Personalization encompasses material selection, membrane choice, surgical approach (lateral vs. transcrestal), and timing of implant placement relative to graft maturation [53,54,55,56]. Long-term implant survival in grafted sinuses exceeds 95% [57].

#### 3.3.5. Autologous Biological Adjuvants (PRF)

Platelet-rich fibrin promotes angiogenesis, growth factor release, and alveolar bone regeneration [58]. The evidence on PRF in augmentation procedures is heterogeneous [◑]. While some studies report accelerated early osseointegration and higher ISQ values at weeks 1 and 4 [59], multiple RCTs have found no statistically significant difference in implant survival or bone gain at 12–36 months compared with unsupplemented protocols. PRF may provide the most meaningful benefit in patients with compromised healing biology (diabetes, osteoporosis, smoking); this patient-specific benefit has not been established in adequately powered prospective trials. A systematic review by Ivanovski et al. confirms the pro-osseointegration effect of autologous platelet concentrates [60]. Its inclusion in the Personalized pillar is therefore conditional on patient-specific biological indication rather than routine application.

#### 3.3.6. Metabolic and Pharmacological Personalization 

Metabolic and pharmacological personalization represents the dimension most closely aligned with precision medicine in its original sense: (1) vitamin D optimization: correction of deficiency (target 25-OH-D > 30 ng/mL) before implant placement in at-risk patients [◑]; (2) bisphosphonate management: individualized drug holiday assessment guided by bone turnover markers [◑]; (3) statin consideration: emerging evidence of a pro-osseointegrative effect in high-risk patients [○]; and (4) individualized antibiotic prophylaxis based on systemic risk profile and local resistance patterns [◑].

### 3.4. Participatory Implantology

#### 3.4.1. Shared Decision-Making

Shared decision-making (SDM) is the collaborative process by which clinicians and patients integrate clinical evidence with individual values and preferences [61]. Recent evidence reveals a systematic gap between the SDM patients desire and what clinicians provide [62,63]. Validated prospective facilitation tools include the Option Grid (structured comparative table of options in patient-accessible language) and question prompt lists [64]. The SDM-Q-9 is a retrospective psychometric instrument designed to measure the extent to which the patient felt they participated in the decision after the consultation; it is not a prospective facilitation tool and should not be administered during the decision-making process itself [65]. In Table 2, the SDM-Q-9 has been relocated to an outcome-assessment row accordingly. True SDM before implant placement must include explicit presentation of the long-term financial burden of lifelong SPiT as an integral component of treatment cost. When predictive stratification classifies a patient as very high risk and the patient, fully informed, elects to proceed, the 4P framework does not override clinician judgment: predictive scores are inputs to the shared decision, not its determinant; documentation of risk communication is essential.

#### 3.4.2. Patient-Reported Outcome Measures

The OHIP-14 is the most widely validated PROM in implant dentistry [66,67]. A recognized limitation is its insensitivity to early asymptomatic peri-implant disease: peri-implant mucositis and early peri-implantitis do not measurably impair quality of life until advanced stages. OHIP-14 must therefore be supplemented by clinical and radiographic parameters for biological monitoring, and ideally by biomarker surveillance. Development of peri-implant disease-specific PROMs with sensitivity to early tissue changes represents an unmet research need. The 4th EAO Consensus Conference recommended integration of PROMs into implant outcome assessment [68,69] and SDM tools improve patient satisfaction and reduce anxiety [70].

#### 3.4.3. Therapeutic Education and Remote Monitoring

Therapeutic patient education (TPE) encompasses interdental hygiene technique training, understanding of early disease warning signs, knowledge of recall protocol rationale, and identification of modifiable behavioral risk factors. Digital tools—smartphone-based coaching applications, remote intraoral camera monitoring, and teledentistry platforms—expand the reach of participatory care between professional visits [71,72].

#### 3.4.4. The 4P Algorithm: Integrating Participation Across the Treatment Continuum

**Figure 2:**
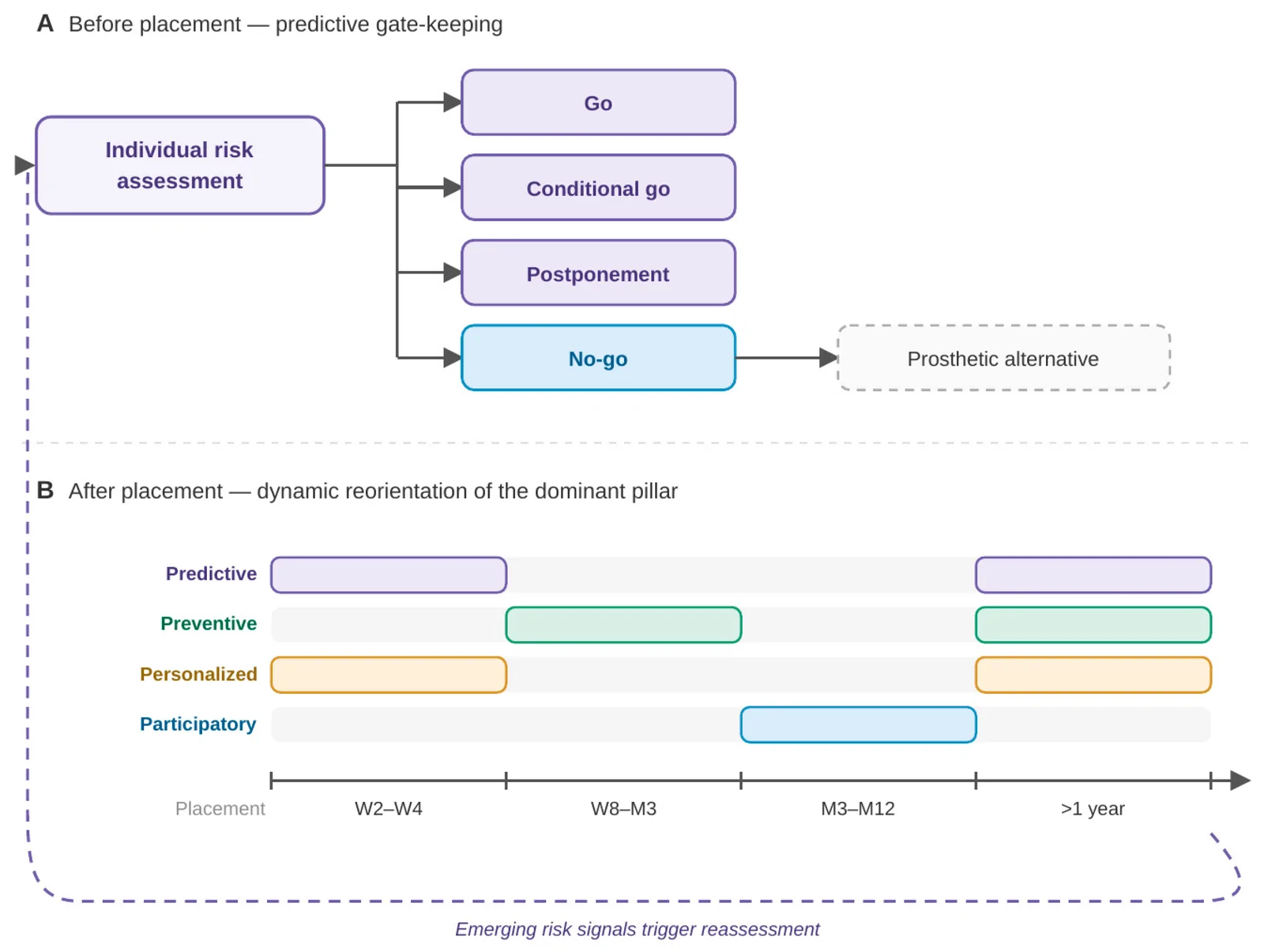
Sequential logic of the 4P model (conceptual illustrative framework). (**A**) Before implant placement, individual risk assessment governs a gate-keeping decision. (**B**) After placement, the predominant pillar shifts along the postoperative timeline as new risk signals emerge; shaded bands indicate proposed clinical emphasis, not exclusion of other pillars. The Preventive pillar begins at day 0. Arrows indicate the sequential decision pathway; the dashed loop indicates that emerging signals trigger reassessment of individual risk. Color coding distinguishes the four pillars. Abbreviations: M, month after placement; W, week after placement.

Table 2 presents the integrated 4P clinical algorithm organized by treatment phase and Table 3 presents the sequential 4P decision framework, distinguishing the pre-placement risk stratification phase (go/no-go decision) from the post-placement dynamic pillar reorientation phase according to emerging risk signals (Figure 2). The pillar predominance indicated in Figure 2 represents proposed clinical emphasis within a conceptual illustrative framework; it does not imply the absence or lesser importance of the other pillars during any given interval.

**Table 2:** Integrated 4P clinical algorithm by treatment phase. ✓ Established; ◑ Promising; ○ Investigational.

Phase	Predictive	Preventive	Personalized	Participatory
Preoperative	CBCT bone density mapping ✓; systemic risk factor profiling ✓; radiomic biomarkers ◑; genomic profiling ○	Phase I periodontal treatment ✓; smoking cessation ✓; glycemic control ✓; STH and keratinized mucosa assessment ✓	Prosthetically driven 3D planning ✓; implant surface selection ✓; surgical guide fabrication ✓	Option Grid, question prompt list, active informed consent ✓; elicitation of patient preferences ✓; presentation of long-term maintenance cost ✓ [SDM-Q-9 → relocated to outcome-assessment row]
Perioperative	ISQ at placement ✓; insertion torque ✓	PRF ◑; keratinized mucosa graft if needed ✓; guided bone regeneration if required ✓	Static, dynamic, or robotic guided surgery ✓; antagonist assessment before immediate loading ✓; loading protocol individualized by ISQ and risk profile ✓	Structured postoperative education; implant-specific hygiene instructions
Follow-up	Serial ISQ at W8, M3, M6 ✓; calibrated radiographs ✓; aMMP-8 chairside ◑; RANKL/OPG ratio ◑	SPiT at IDRA-stratified intervals ✓; supramucosal and submucosal debridement ✓	Occlusal adjustment ✓; prosthetic optimization by CAD/CAM ✓	PROMs (OHIP-14) ✓; patient education; remote monitoring ◑
Outcome assessment				SDM-Q-9 ✓ [retrospective—measures decision quality perceived by patient after consultation; NOT a prospective facilitation tool]

CAD/CAM, computer-aided design/computer-aided manufacturing; IDRA, Implant Disease Risk Assessment; ISQ, implant stability quotient; OHIP-14, Oral Health Impact Profile-14; PRF, platelet-rich fibrin; PROMs, patient-reported outcome measures; SDM-Q-9, 9-item Shared Decision Making Questionnaire; SPiT, supportive peri-implant therapy; STH, supracrestal tissue height.

**Table 3:** Sequential 4P decision framework. Conceptual framework—not a prospectively validated protocol.

Phase/Timepoint	Determinant	Decision or Clinical Action	Priority 4P Pillars
A. Before implant placement: risk stratification and go/no-go decision			
Low risk	No systemic or local contraindication	Go: standard protocol	Predictive; Preventive; Personalized; Participatory
Moderate risk, site-related	Bone deficit, insufficient STH or keratinized mucosa, complex anatomy	Conditional go: augmented protocol (STH graft, bone augmentation)	Personalized; Predictive
Moderate risk, patient-related	History of periodontitis, heavy smoking (>10 cig/day), uncertain compliance, borderline HbA1c (7–8%)	Conditional go: prehabilitation programme; reassessment at 3–6 months	Participatory; Preventive; Predictive
High risk	Cumulative uncontrollable risk: uncontrolled diabetes, recent radiotherapy, untreated active periodontitis	Postponement: mandatory prior treatment; reassessment at 3–6 months	Predictive; Participatory
Very high risk	Severe uncontrolled systemic disease; permanent absolute contraindication	No-go: if contraindication is modifiable, prehabilitation + reassessment; if permanent, prosthetic alternatives discussed via SDM with explicit acknowledgment that systemic/compliance issues also affect prognosis of alternative therapies	Participatory; Predictive
B. After implant placement: dynamic reorientation of the dominant pillar			
W2–W4	ISQ < 60 at placement, or declining trend	Delay loading; non-invasive clinical monitoring at W2 and W4 (percussion test, mucosal healing assessment) WITHOUT abutment removal; ISQ re-measurement at W8 only when mucosal seal is established	Predictive; Personalized
W8–M3	Incipient peri-implant mucositis (BOP without bone loss)	Immediate nonsurgical debridement; hygiene reinforcement; recall at 3 months	Preventive
M3–M12	Poor compliance; degraded PROMs (rising OHIP-14)	Motivational interviewing; revised maintenance schedule; remote monitoring	Participatory
Beyond 1 year	Confirmed peri-implantitis (bone loss > 2 mm, PD ≥ 6 mm)	PISF biomarker panel ◑; IDRA reclassification ✓; surgical treatment ✓	Predictive; Preventive; Personalized

Note: Genomic profiling is not included as a decision criterion given the current absence of prospective multicenter validation for clinical decision-making. BOP, bleeding on probing; HbA1c, glycated hemoglobin; IDRA, Implant Disease Risk Assessment; ISQ, implant stability quotient; M, month; OHIP-14, Oral Health Impact Profile-14; PD, probing depth; PISF, peri-implant sulcular fluid; PROMs, patient-reported outcome measures; SDM, shared decision-making; STH, supracrestal tissue height; W, week. Pillar predominance represents proposed clinical emphasis, not exclusion of other pillars.

## 4. Discussion

The 4P framework applied to implant dentistry represents a paradigmatic shift from a procedure-centered to a patient-centered model. The sequential decision-making logic distinguishing pre-placement gate-keeping from post-placement dynamic pillar reorientation constitutes a novel operational contribution, presented here as a conceptual framework rather than a prospectively validated protocol.

The concept of prehabilitation formalizes pre-existing Phase I periodontal obligations into a structured multidomain timeline—its contribution is organizational, not the introduction of new therapeutic actions. The distinction between EIL and peri-implantitis as biologically distinct entities is clinically consequential: ISQ monitoring without abutment removal at W2–W4 addresses EIL surveillance; PISF biomarker monitoring and IDRA-stratified SPiT address peri-implantitis prevention.

AI predictive models carry a convergent limitation: most are monocentric, retrospective, and lack prospective external validation. We propose a tiered implementation framework: (1) minimum viable 4P model—accessible in any implantology practice: clinical risk assessment, IDRA, OHIP-14, Option Grid, IDRA-stratified recall; (2) enhanced model—for specialist centers: digital workflow, dynamic guidance, chairside aMMP-8; (3) research-stage model—for academic settings: genomics, radiomics, digital twin. The framework should be interpreted as a structured reasoning architecture rather than a fixed protocol.

## 5. Conclusions

The 4P framework provides implant dentistry with a long-needed conceptual model for navigating the transition from a procedure-centered to a patient-centered discipline. Regular professional maintenance is associated with a peri-implantitis frequency of 18% versus 43.9% in non-compliant patients [39] illustrating the measurable clinical impact of one component of the preventive pillar; this finding derives from a single cohort analysis and cannot be presented as evidence of the integrated 4P model’s efficacy. The sequential decision-making framework distinguishing pre-placement gate-keeping from post-placement dynamic pillar reorientation represents an additional operational contribution.

Future research should prioritize: (1) prospective multicenter validation of AI predictive models and genomic risk markers; (2) RCTs evaluating the combined impact of integrated 4P protocols on peri-implant health and patient-reported outcomes; (3) development of explainable AI architectures for implant dentistry; (4) implementation science studies evaluating feasibility, equity, and cost-effectiveness across resource levels; and (5) formal evaluation of tiered implementation models in primary care settings.

## Figures and Tables

**Table 1:** Pre-implant risk factors and corresponding management strategies.

Risk Factor	Action Before Implant Placement
Smoking	Smoking cessation recommended (≥4 weeks before surgery)
Diabetes	Glycemic control before surgery (HbA1c < 7% desirable; >8% recognized systemic risk factor—AO/AAP 2025 [12])
Active periodontitis	Mandatory Phase I periodontal treatment; elimination of residual pockets with BOP before implant placement
Poor oral hygiene	Patient education, motivation, and verification of plaque control before any intervention
Insufficient keratinized mucosal width (<2 mm)	Connective tissue graft or free gingival graft at or before implant placement
ADDED—Thin supracrestal tissue height (STH < 3 mm)	Consider subcrestal implant positioning or connective tissue augmentation to achieve STH ≥ 3 mm
Bruxism	Occlusal splint; occlusal rehabilitation if required; relative contraindication to immediate loading
Head and neck radiotherapy	Individualized protocol with oncology team; site- and dose-specific assessment

BOP, bleeding on probing. STH, supracrestal tissue height. HbA1c thresholds derived from AO/AAP consensus [12].

## Data Availability

No new data were created or analyzed in this study. All data discussed are available in the published sources cited in the reference list.
